# Accelerated Growth Rate Induced by Neonatal High-Protein Milk Formula Is Not Supported by Increased Tissue Protein Synthesis in Low-Birth-Weight Piglets

**DOI:** 10.1155/2012/545341

**Published:** 2012-01-24

**Authors:** Agnès Jamin, Bernard Sève, Jean-Noël Thibault, Nathalie Floc'h

**Affiliations:** ^1^INRA, UMR1348, PEGASE, 35590 Saint-Gilles, France; ^2^INRA, Agrocampus Ouest, UMR1348, PEGASE, 35000 Rennes, France; ^3^INSERM U699, 75018 Paris, France

## Abstract

Low-birth-weight neonates are routinely fed a high-protein formula to promote catch-up growth and antibiotics are usually associated to prevent infection. Yet the effects of such practices on tissue protein metabolism are unknown. Baby pigs were fed from age 2 to 7 or 28 d with high protein formula with or without amoxicillin supplementation, in parallel with normal protein formula, to determine tissue protein metabolism modifications. Feeding high protein formula increased growth rate between 2 and 28 days of age when antibiotic was administered early in the first week of life. This could be explained by the occurrence of diarrhea when piglets were fed the high protein formula alone. Higher growth rate was associated with higher feed conversion and reduced protein synthesis rate in the small intestine, muscle and carcass, whereas proteolytic enzyme activities measured in these tissues were unchanged. In conclusion, accelerated growth rate caused by high protein formula and antibiotics was not supported by increased protein synthesis in muscle and carcass.

## 1. Introduction

Formulation of artificial milk for the neonate has improved in the last decade. While breast feeding is encouraged, high protein formulas are still used in at-risk populations such as low-birth-weight (LBW) babies who have suffered intra-uterine growth restriction (IUGR) to ensure a rapid postnatal catch-up growth and brain development [1]. This nutritional practice is based on the anabolic effect of high-protein formulas described in young animals born with normal birth weight, attributed to the stimulatory effect of amino acids on muscle and liver protein synthesis [2]. However, no such anabolic effect have ever been reported in LBW neonates. Owing to the high vulnerability of LBW neonates, antibiotic treatments are frequently associated with HP formulas to prevent infection from the mother, and associated septicemia. In this work, we also studied the effect of antibiotic treatment administration associated with an HP formula on tissue protein metabolism. Amoxicillin was selected because this antibiotic is widely used in neonates to prevent severe infection caused by group B streptococci. It can be administered orally to reach effective plasma amoxicillin concentrations in human neonates [3]. When supplied orally, this antibiotic may also interfere with the effects of high-protein supply on the development of microbiota [4] and may decrease the risk of digestive disorders.

Animal models fed and reared in a well-controlled environment can be used to test nutritional strategies and study their impact on the development of LBW neonates. The LBW pig is a natural model similar to human asymmetric IUGR [5]. In this species, growth restriction occurs naturally and is caused mainly by spontaneous placental vascular insufficiency [6]. The *in utero *growth restriction preferentially concerns the muscles, followed by intraabdominal organs, such as the gastrointestinal tract, kidneys, and liver, whereas brain development is protected [7, 8]. In addition, the similar body protein composition reported in the pig and human fetus indicates that the pig is also a suitable model for studying protein nutrition and metabolism in neonates [9].

Our objective was to determine whether feeding low-birth-weight piglets a HP formula with or without an added antibiotic, in comparison with a normal protein (NP) formula, could modify tissue protein metabolism. The NP formula was made up to resemble sow milk, while the HP formula contained 50% more protein. We measured, at fed state, both protein synthesis and activities of enzymes involved in proteolysis in the jejunum, ileum, liver, semitendinosus muscle, and eviscerated carcass of artificially reared piglets. Protein synthesis rate was evaluated after piglets were injected with a flooding dose of l-[^15^N]-valine. We also measured the activities of the three main enzymatic systems involved in tissue protein breakdown: the lysosomal, the Ca^2+^-activated, and the ATP-ubiquitin-dependent pathways, that is, cathepsin L, calpain, and proteasome respectively. Additionally, we investigated the effect of duration of high-protein formula feeding during the neonatal period on growth and tissue protein metabolism. For this purpose, piglets were fed the high protein formula from d2 to d7 or from d2 to d28: antibiotics were delivered orally from d2 to d7.

## 2. Materials and Methods

### 2.1. Animals, Allotments, and Experimental Design

Experimental procedures and animal care were compliant with the French legislation in force. Authorization to experiment and practice surgery on living animals was granted by the French Ministry of Agriculture (certificate no. 7719). A total of 36 full-term crossbred (Piétrain × (Large White × Landrace)) piglets were obtained from the experimental herd of INRA (Saint-Gilles, France). Piglets were selected from among 18 litters with a piglet mean birth weight of 1.30 ± 0.03 kg, close to the mean birth weight in the population of the INRA herd, based on the past 10 years (1.44 ± 0.28, based on 582 litters, [10]). Within each litter, all piglets were weighed at birth and piglets were selected, irrespective of sex, when their birth weight was around the 10th percentile of the birth weight of the herd, that is, 0.96 ± 0.11 kg. Piglets whose birth weight was below the 5th percentile were not viable and so were not selected. After 2 days of colostrum intake, piglets were separated from the sows and 18 pairs of littermate piglets with similar body weight were formed to compare the three experimental treatments in a balanced incomplete block design where one treatment was compared with another within a pair of piglets: NP versus HP, NP versus HPA, and HP versus HPA (HP formula, supplemented with amoxicillin). Each comparison was repeated six times for piglets slaughtered at d7 and d28 as shown in Table 1. This experimental design was used to overcome the difficulty of comparing the three treatments between littermate piglets with similar body weight. Here, the litter effect is considered as a random effect (residual error term) and the estimates are a function of treatment effects and within litter error only.

All the piglets were placed in incubators (33°C, 60% humidity) and bottle-fed 10 times a day at 2 h intervals between 07:00 and 23:00, plus an extra meal at 03:00 between d2 and d7. At d7, piglets were transferred into individual cages equipped with an automated device delivering milk 10 times a day (same schedule) in a temperature-controlled room at 30°C until d28. Clinical observations were recorded daily. Piglets were weighed daily from d2 to d6, then weekly until d28. Feed intake was recorded daily.

### 2.2. Animal Feeding

The individual daily milk quantity delivered to the piglets was calculated according to their body weight, and following a predefined feeding plan based on milk intake of suckling piglets [11]. Briefly, piglets received 1305 kJ/kg of their metabolic weight (body weight^0.75^) per day between d2 and d28. The NP formula was calculated to supply the same proportions of protein, fat, and carbohydrates as in sow milk [11]. The casein : whey protein proportion was also adjusted to average values reported for sow milk at 46 : 54 [12]. The HP formula was designed to provide a 50% higher amount of protein of similar composition from d7 to d28. From d2 to d7, the amount of protein offered to the HP piglets was only 35% higher than that offered to the NP piglets to prevent metabolic disturbances due to a high-protein intake, as previously described [13]. This was achieved by offering the HP piglets a milk formula prepared by mixing NP and HP formulas (30/70 v/v). The HPA piglets were fed the HP diet as previously described. Between d2 and d7 they also received 50 mg/kg/d of amoxicillin in meals three times a day at 09:00, 15:00, and 21:00. With this dose of amoxicillin delivered orally, plasma amoxicillin concentration, determined during a preliminary trial (data not shown), was close to concentrations measured in babies receiving amoxicillin intravenously. Both NP and HP milk formulas were manufactured by the Laiterie de Montaigu (Montaigu, France) and are described in Table 2. Milks were prepared each day for the next day by dissolving and homogenizing appropriate amounts of HP and NP powders with water to reach 200 g/L. Before the age of 7 days bottles were immediately filled, kept at 4°C, and then warmed up and rehomogenized before meals. After age 7 days the daily ration of NP or HP milk for each pig was placed in the refrigerated compartment of the individual cage, with stirring, and automatically delivered as described above.

### 2.3. Protein Synthesis Measurements

Two hours after the last meal, piglets were anesthetized by inhalation of sevofluorane (Baxter, Maurepas, France) using a facemask, and a catheter was inserted into the jugular vein. Fractional protein synthesis rate was measured in anesthetized piglets according to the flooding dose procedure [14]. Briefly, a solution of l-[^15^N]-valine was prepared by mixing 20% of l-[^15^N]-valine (99% mol percent excess; Tracer technologies, Sommerville, MA) with 80% of unlabelled l-valine to obtain a final enrichment of 19.8 mol percent excess. Valine was diluted in water to give a final concentration of 150 mmol/L, and 1.5 mmol/kg BW was injected into the piglets through the jugular catheter. Two milliliters of blood was collected in heparinized ice-cooled tubes just before the injection, and then 7 and 14 minutes after the injection. Blood was centrifuged, and plasma was stored at −20°C until analysis. Fifteen minutes after the valine injection, piglets were sacrificed with a lethal dose of T61 (Intervet, Beaucouzé, France) and exsanguinated. Samples of liver and semitendinosus muscle were immediately collected and frozen in liquid nitrogen. The gastrointestinal tract was quickly removed, dissected free of mesenteric attachment, and placed on a smooth, cold-surfaced tray. Rapidly, one 15 cm segment corresponding to the proximal jejunum was collected from the Treitz ligament, and another one 15 cm long before the ileocaecal junction corresponding to the distal ileum. All the segments were emptied and rinsed with saline buffer. Five centimeter lengths of each segment were quickly frozen in liquid nitrogen for protein synthesis analyses. The other 10 cm of the mucosa was scraped and frozen in liquid nitrogen for proteolysis enzyme activity analyses. Whole eviscerated carcasses were frozen in liquid nitrogen. All the tissues were sampled and frozen following the same sequence, that is, liver, muscle, small intestine, and carcass. Hence for each tissue, the time before freezing was similar between pigs. The duration of anesthesia never exceeded 30 min and the piglets lay on a warm cloth throughout the procedure. Given these conditions we assumed that the effect of anesthesia on the measured criteria was only slight.

The isotopic enrichment of l-[^15^N] valine in the plasma free pool (*S*_*A*_) and protein-bound in the tissues (*S*_*B*_) was determined as previously described [15]. The fractional synthesis rate or *K*_*s*_, corresponding to the percentage of protein mass synthesized per day, was calculated from *K*_*s*_(%/day) = (*S*_*B*_ × 100)/(*S*_*A*_ × *t*), where *t* is the l-[^15^N]-valine incorporation time (time elapsed between injection of the flooding dose of valine and sacrifice of the animals). Protein concentrations were measured as described [16] and RNA concentration was quantified by spectrophotometry (Nanodrop, Wilmington, USA). Ribosomal capacity for protein synthesis (*C*_*s*_) was used as an indicator of cellular potential for protein synthesis and calculated as the ratio of tissue RNA to protein content (mg/g). Finally the efficiency of protein synthesis (*K*_RNA_) was evaluated as the amount of protein synthesized per ribosomal RNA (g protein synthesized/day/mg RNA) according to *K*_RNA_ = (*K*_*s*_ × 10)/*C*_*s*_.

### 2.4. Measurement of Proteolysis Enzyme Activities

The activities of proteasome, calpain (1 and 2), and cathepsin L in tissues were determined as described previously [17]. Five hundred milligrams of each tissue were homogenized (Kinematica, Polytron, Littau-Lucerne, Switzerland) in ice-cold lysing buffer containing 30 mM Tris-HCl (pH 7.2), 1 mM dithiothreitol, 1% Triton X-100 and then centrifuged at 12,000 g for 15 min at 4°C. Protein concentration was then measured in supernatants by the Bradford method [18]. For proteasome and calpain activities, 10 *μ*L of diluted supernatants was incubated with 100 *μ*L of the fluorogenic proteasome substrate Suc-LLVY-MCA at 70 *μ*M (Calbiochem, Darmstadt, Germany) for 90 min at 37°C. For each sample, incubation was also performed in the presence of specific inhibitors: clasto-lactacystin *β*-lactone at 10 *μ*M (Calbiochem) and MDL-28170 calpain inhibitor at 2.5 *μ*M (Biomol Research Laboratories, Plymouth, PA, USA) for proteasome and calpain, respectively. Cathepsin L activity was measured in diluted supernatant using Cathepsin L activity assay kit (Biovision, Mountain View, CA, USA). Two microliters of Ac-FR-AFC substrate at 10 mM was diluted in 50 *μ*L of reaction buffer and incubated with 50 *μ*L of diluted supernatant for 90 min at 37°C. For each sample, incubation was also performed in the presence of 0.4 *μ*L of the specific kit inhibitor at 1 mM. Measurements of proteolysis (unquenched MCA peptide for proteasome and calpain, and released AFC peptide for cathepsin L, resp.) were carried out in a Mithras LB 940 microtiter plate fluorimeter (Berthold Technologies, Thoiry, France; excitation 355 nm; emission 460 nm for proteasome and calpain, and 535 nm for cathepsin, resp.). All activity values were calculated by subtracting the fluorescence obtained in the presence of the specific inhibitor from the value in its absence. Activities were expressed in relative fluorescence units (RFU)/min/mg protein.

### 2.5. Biochemical Tissue and Plasma Composition

Tissues were homogenized in cold water (Kinematica, Polytron) to measure their total protein concentrations. Liver and muscle dry matter was obtained after 2 days at 103°C.

Plasma free amino acid concentrations were measured by UPLC/MS according to Mass Trak Amino Acid Analysis Solution (Acquity system, Waters, Guyancourt, France).

### 2.6. Statistical Analyses

The experimental unit was the piglet. Statistical analysis was performed using the General Linear Model procedure of the Statistical Analysis System (SAS Institute, Cary, NC, USA). The model included the effects of diet and age, with *t*-test as multiple comparisons when appropriate. Results were expressed as least square means and pooled SEM for each age.

## 3. Results

### 3.1. Feed Intake, Body, and Tissue Growth

NP, HP and HPA IUGR piglets had similar birth weights (0.94 kg, 0.95 kg and 0.96 kg resp., pooled SEM = 0.12). Both slaughtered weight and average body weight gain were higher at d28 than at d7 (Table 3). HPA pigs tended to be heavier and grew faster than HP pigs at d28 (*P* < 0.1). When expressed relative to metabolic weight, neither energy nor protein intakes varied in the course of the experiment. As expected, protein intake of HP and HPA piglets was higher than in NP piglets (*P* < 0.001 and < 0.01, at d7 and d28, resp.). Feed conversion, that is, feed intake relative to weight gain ratio, decreased with age (*P* < 0.01) for all three groups. At d7, HPA piglets had higher feed efficiency than HP piglets, but there was no effect of treatments at d7.

When expressed relative to body weight, liver and carcass weight decreased with age (*P* < 0.001, Table 4) while muscle weight did not vary. Liver and muscle weights were unchanged by the dietary treatments at both d7 and d28. Carcasses of HPA piglets were heavier than those of NP piglets at d7 but not at d28. Dry matter content of the liver was higher in HP than in HPA and NP at d7 and d28. Muscle dry matter content increased with age (*P* < 0.001) and there was no significant effect of the dietary treatments.

### 3.2. Tissue Protein Synthesis Rate, Protein Synthesis Efficiency, Ribosomal Capacity, and Protein Content

Protein synthesis rate (*K*_*S*_) and efficiency (*K*_RNA_), ribosomal capacity (*C*_*S*_), and protein content are presented in Table 5. Protein synthesis rate decreased with age in all tissues except the proximal jejunum. This was associated with a decrease in both *C*_*S*_ and *K*_RNA_ in the ileum and liver and *C*_*S*_ in the muscle and the carcass.

At d7, but not at d28, HP formula increased ileum and liver *K*_*S*_ and *K*_RNA_ compared with NP, but there was no difference between NP and HPA. Tissue protein content and *C*_*S*_ did not differ between the different formulas at d7. At d28, *K*_*S*_ was lower in HP than in NP in the jejunum and lower in both HP and HPA than in NP in the carcass. In the carcass, *C*_*S*_ was also lower in HP and HPA compared with NP. In the liver, *C*_*S*_ was lower in the NP group than in HP and HPA, but neither *K*_*S*_ nor *K*_RNA_ was different across the three groups. Both HP and HPA increased liver protein content measured at d28 (*P* < 0.05).

### 3.3. Tissue Activities of Proteolytic Enzymes

Proteasome, calpain and cathepsin L activities are presented in Table 6. The activities of the three enzymes and that of calpain decreased between d7 and d28 in the muscle and liver, respectively. Calpain and cathepsin L activities increased with age in the jejunum but only that of calpain in the ileum. At d7, there was no effect of formula on proteolytic activities. At d28, liver proteasome and calpain activities were higher in both HP and HPA groups than in the NP group.

### 3.4. Plasma Amino Acid, Urea, and Ammonia Concentrations

Plasma concentrations of lysine, methionine, and tryptophan, among essential amino acids, and of citrulline, glycine, and ornithine among nonessential amino acids, increased between d7 and d28, whereas those of alanine, serine, proline, and taurine decreased (Table 7). At d7, plasma concentrations of almost all essential amino acids, except phenylalanine and histidine, and those of serine, were higher in pigs fed HP and HPA formulas than in those fed the NP formula. Lysine plasma concentrations were still higher in HP and HPA groups than in the NP group at d28. The same effect was observed for taurine, NH_3_, and urea, whereas concentrations of histidine, glutamic acid, glycine, and serine were higher in NP than in HP and HPA groups.

## 4. Discussion

High-protein milk is usually given to LBW neonates to promote catch-up growth [1]. This study shows that feeding neonatal LBW piglets with HP formulas increased growth rate between 2 and 28 days of age compared with those fed NP formula when an antibiotic has been administered early during the first week of life. This may be because diarrhea occurred from d7 to d15 in HP piglets, but not in HPA piglets. Higher growth rate in HPA piglets was associated with higher feed conversion and more surprisingly with reduced protein synthesis rate in the small intestine, muscle and carcass, whereas proteolytic enzyme activities measured in these tissues were unchanged. Liver protein synthesis and protein contents were increased in HP compared with NP and HPA piglets at d7 and d28, respectively, whereas proteasome and calpain activities were reduced at d28 in HP and HPA compared with NP piglets.

As previously reported in neonatal normal birth-weight piglets [2, 19–21], protein synthesis rate decreased between d7 and d28 in almost all the tissues examined, except in the jejunum. Levels of mRNA coding for proteolytic enzymes and their proteomic expression had been investigated in normal-weight piglets fed different diets [21–23], and in those suffering from IUGR [24], but activities had never been reported in neonatal piglets. The highest activities were found in tissues with the highest protein synthesis rate, namely, the liver and small intestine, while the lowest activities were reported in muscle. Surprisingly, the activity of cathepsin L was highest in muscle. In this tissue, lysosomal proteases are abundant during fetal development but not in adults [25]. The high cathepsin L activity reported in our experiment could be attributed to the muscle immaturity of neonatal LBW piglets, supported by the decrease in cathepsin L activity between ages 7d and 28d. In the small intestine of LBW piglets, calpain and cathepsin L activities increased with age, while proteasome activity remained unchanged. In both small intestinal segments, that is, jejunum and ileum, proteasome activity was three times higher than calpain and cathepsin L activities. Such differences between the activities of the enzymes of the three proteolytic pathways have also been reported in adult rats [26]. Calpain is involved in cytoskeleton remodeling. It regulates brush border microvilli assembly during differentiation and microvili effacement caused by *Escherichia coli* [27]. Increased calpain activity with age may be involved in enhanced brush border turnover in response to gut colonization by bacteria and to the presence of exogenous dietary components in the lumen. Increased cathepsin L activity between d7 and d28 may be associated with a decreased flux of macromolecules across the intestinal barrier during the neonatal period [28].

To our knowledge, our study is the first to assess the impact of dietary protein content on both protein synthesis and the enzyme activities of the components of the three main proteolytic pathways in different tissues of LBW piglets. In these, in contrast to normal-weight piglets [2], high protein intake increased both *K*_*S*_ and *K*_RNA_ in the ileum at d7. For an unknown reason, amoxicillin seems to prevent this effect. We speculate that protein synthesis in the jejunum increases in response to an excessive proliferation of microbiota caused by high-protein intake [4] and that the antibiotic reduced this proliferation. High protein intake stimulated liver protein synthesis at d7 but not at d28. Our results are in agreement with previous data [2] obtained in normal-weight piglets aged 7 days. In this experiment, the stimulatory effect of protein was much more marked in overnight-fasted than in fed piglets when protein synthesis was measured. This suggests that in the present experiment, the liver of fed LBW piglets responded to a high-protein supply comparably to that of fasted normal-weight piglets. Liver protein synthesis stimulation by amino acids was lost during the onset of the postnatal period [20], consistent with the lack of stimulatory effect of high protein diet at d28. In rats, high amino acid intake exerts an anti-proteolytic effect [29]. Our results show that liver proteasome and calpain activities measured at d28 were reduced by HP formula. This result indicates that unlike protein synthesis, the nutritional control of liver proteolysis may increase with age.

The issue of optimal protein intake in low-birth-weight infant formula remains controversial. Studies in IUGR infants showed that high-protein intake increased protein balance [1]. In pigs and other species, feeding HP diets is suspected to induce metabolic disorders, particularly in preterm or IUGR neonates [13, 30]. However, experimental data showed no evidence of acute toxicity with high enteral and parenteral amino acid intake [31], as indicated by our data showing no excessive accumulation of urea, ammonia, or free amino acids in the plasma. Amino acids, especially essential amino acids, accumulated in the plasma with both HP formulas at d7 and much less at d28. This may reflect a better utilization of additional amino acids for protein synthesis at d28 and/or a more efficient degradation of amino acids in excess. Thus our data indicate that high-protein intake did not increase growth rate of LBW when delivered briefly, that is, d2 to d7. However, as previously reported [32], growth rate was accelerated when HP diet was delivered until d28. Our data reported a similar effect, but only in HPA piglets, which did not suffer from diarrhea, unlike HP piglets. High protein-feeding accelerated the colonization of the gut by bacteria [4], and this may represent an increasing risk of developing gut disorder in young and immature neonate piglets. In the present experiment, amoxicillin delivered orally probably helped to prevent diarrhea by controlling bacteria proliferation. Accelerated growth rate was not supported by increased tissue protein synthesis rate and decreased proteolytic activities, except in the liver, while feed conversion was improved. This last finding is supported by results showing a reduction in adipose tissue development in LBW piglets fed a high-protein diet from age 2 to 28 days [32]. Liver protein metabolism seems to be a key part of the response of LBW piglets to high-protein intake. Because this organ plays a central role in the interplay between protein and energy metabolism, its role deserves further study in relation with nutrient partitioning for growth and development.

Sow milk contains 5.5 times more protein and 1.4 times less carbohydrate than human milk. Although differences between species must preclude any direct extrapolation of our findings to human infants, their relevance to the use of HP formulas in LBW calls for discussion. In neonatal LBW piglets, the tested HP and HPA formulas were not harmful in the short term, at least on the basis of tissue protein metabolism. The question is whether or not it may have long-term consequences in adulthood: HP diet delivered during the postnatal period may lead to deterioration of renal function in adult rats [33]; decrease in insulin sensitivity has been reported in one-year-old LBW pigs [34], and this may promote diabetes and obesity as described in human and rodent adults after neonatal enriched diet feeding [35]. Further studies are thus needed to investigate long-term effects of such HP diet in our LBW piglet model. The potential relevance of these findings calls for a critical examination of the responses of protein metabolism in various tissues and their impact on short-term development with regard to the usual HP formula strategies used in LBW infants.

## Figures and Tables

**Table 1:** Experimental incomplete block design.

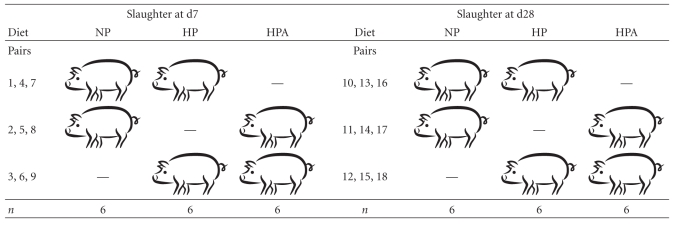

**Table 2:** Milk composition.

	NP	HP
Ingredients	g/100 g powder	
Vegetable oil mix	24.8	22.2
Lactose	19.5	21.3
Milk fat	14.5	12.9
Whey protein concentrate	14.2	20.3
Skimmed milk powder	8.8	—
Potassium caseinate	8.4	14.7
Tricalcium phosphate	2.6	2.4
l-proline	1.7	1.4
l-glutamic acid	0.9	0.9
Arachidonic acid	0.9	0.8
l-arginine	0.8	0.6
l-glycine	0.6	0.4
Vitamin and mineral premix^1^	0.5	0.5
Dicalcium phosphate	0.5	0.3
l-valine	0.3	0.3
l-histidine	0.2	0.2
Magnesium chloride	0.2	0.2
l-cysteine	0.1	0.1
DHA-enriched fish oil	0.2	0.2
Choline ditartrate	0.2	0.2
Composition		
Energy (kJ/L)	4753	5030
Protein (g/L)	51.4	77.0
Fat (g/L)	82.0	80.0
Carbohydrate (g/L)	49.0	47.0
Dry matter (g/L)	196.0	223.5

^1^Trace mineral and vitamin premix supplied per 100 g of powder: retinol 0.3 mg, cholecalciferol 0.005 mg, *alpha*-tocopherol 2.0 mg, menadione 0.2 mg, thiamin 0.2 mg, riboflavin 0.5 mg, d-pantothenic acid 0.1 mg, niacin 0.2 mg, d-biotin 0.02 mg, cyanocobalamin 0.003 mg, folic acid 0.1 mg, pyridoxine 0.5 mg, ascorbic acid 4.0 mg, Fe 10.0 mg, Zn 10.0 mg, Mn 4.0 mg, Co 0.1 mg, I 0.06 mg, Se 0.03 mg.

**Table 3:** Growth performance of piglets fed NP, HP and HPA diets at d7 and d28.

	d7					d28					Age effect
	NP	HP	HPA	SEM	*P*	NP	HP	HPA	SEM	*P*	
Final weight	1.44	1.39	1.48	0.71	Ns	5.48^ab^	4.85^b^	6.20^a^	0.41	<0.1	<0.001
Body weight gain, g/d/kg body weight	81.5	70.6	81.7	5.97	Ns	148.6^ab^	127.4^a^	181.5^b^	15.00	<0.1	<0.001
Energy intake, kJ/d/kg metabolic weight	1189.6^a^	1118.7^ab^	1073.4^b^	33.9	<0.1	1095.4	1195.3	974.8	70.69	ns	Ns
Protein intake, g/d/kg metabolic weight	12.9^a^	15.7^b^	15.0^b^	0.43	<0.001	11.8^a^	18.3^c^	14.9^b^	0.94	<0.01	Ns
Feed conversion, g feed/g metabolic weight	0.044	0.053	0.052	0.003	ns	0.042^ab^	0.040^a^	0.046^b^	0.002	<0.1	<0.01

Values are least square means and pooled SEM calculated in pigs slaughtered at d7 and d28, *n* = 6/group. *P* (effect of formula) and age effects are presented. Differences were considered significant when *P* < 0.05 but trends are presented when 0.1 < *P* < 0.05. Means with a different superscript letter differ at *P* < 0.05.

**Table 4:** Relative weight (g/kg body weight) and dry matter (g/100 g tissue) measured in the liver, semitendinosus muscle and eviscerated carcass of fed NP, HP, and HPA diets at d7 and d28.

			d7					d28			Age effect
	NP	HP	HPA	SEM	*P*	NP	HP	HPA	SEM	*P*	
Liver											
Relative weight, g/kg body weight	36.3	33.2	31.2	1.89	Ns	28.3	29.4	27.6	1.04	ns	<0.001
Dry matter content, g/100 g of tissue	26.5^a^	30.3^b^	26.2^a^	1.11	<0.05	27.0^a^	29.2^b^	26.3^a^	0.59	<0.05	ns
Semitendinosus muscle											
Relative weight, g/kg body weight	2.5	2.9	2.7	2.69	Ns	2.5	3.1	2.1	0.31	ns	ns
Dry matter content, g/100 g of tissue	19.5	19.5	19.8	0.61	Ns	23.5	21.3	24.2	1.39	ns	<0.001
Eviscerated carcass											
Relative weight, g/kg body weight	845.1^b^	860.4^ab^	888.5^b^	11.4	<0.05	831.2	834.4	840.1	4.02	ns	<0.001
Dry matter content, g/100 g of tissue	nd	nd	nd	—	—	nd	nd	nd	—	—	—

Values are least square means and pooled SEM calculated in pigs slaughtered at d7 and d28, *n* = 6/group. *P* (effect of formula) and age effects are presented. Differences were considered significant when *P* < 0.05 but trends are presented when 0.1 < *P* < 0.05. Means with a different superscript letter differ at *P* < 0.05. nd: not determined.

**Table 5:** Fractional rates (*K*_*s*_, %/d) of protein synthesis, protein synthesis efficiency (*K*_RNA_), ribosomal capacity (*C*_*S*_) and protein contents (g/100 g tissue) measured in various tissues of piglets fed NP, HP, and HPA diets at d7 and d28.

	d7					d28					Age effect
	NP	HP	HPA	SEM	*P*	NP	HP	HPA	SEM	*P*	
*K*_*s*_, %/d											
Proximal jejunum mucosa	55.5	58.9	57.6	3.11	Ns	60.9^a^	50.6^b^	56.1^ab^	1.71	<0.01	ns
Distal ileum mucosa	58.5^a^	64.2^b^	54.5^a^	2.64	<0.05	53.3	53.5	48.2	2.13	ns	<0.01
Liver	67.5^a^	82.9^b^	75.1^ab^	4.27	0.06	58.7	53.2	52.4	2.51	ns	<0.001
Semitendinosus muscle	18.6	19.7	20.7	1.06	Ns	11.8^a^	10.6^ab^	9.8^b^	0.89	<0.1	<0.001
Carcass	16.2	17.1	18.2	0.91	Ns	14.4^a^	12.5^b^	11.9^b^	0.34	<0.01	<0.001
*K*_RNA_, g/g/d											
Proximal jejunum mucosa	13.5	14.3	14.2	0.67	Ns	14.7	13.3	14.4	0.44	ns	ns
Distal ileum mucosa	12.0^a^	13.3^b^	12.4^ab^	0.46	0.07	12.0^a^	12.4^a^	10.7^b^	0.39	0.06	0.07
Liver	13.6^a^	18.3^b^	16.3^ab^	0.76	<0.01	12.9	12.7	13.7	0.90	ns	<0.01
Semitendinosus muscle	10.8	10.9	11.6	0.79	Ns	11.8	10.6	9.8	0.86	ns	ns
Carcass	7.5	7.9	8.3	0.58	Ns	8.6	8.6	8.3	0.35	ns	ns
*C*_*s*_, mg RNA/g protein											
Proximal jejunum mucosa	41.4	41.2	40.6	167	Ns	41.4	38.0	39.2	1.22	ns	ns
Distal ileum mucosa	48.8	48.8	44.0	1.97	Ns	44.3	43.0	45.5	2.13	ns	0.09
Liver	49.8	45.3	46.5	2.34	ns	45.6^a^	42.4^a^	38.6^b^	1.60	<0.05	<0.01
Semitendinosus muscle	17.4	18.5	17.8	0.94	Ns	10.4	9.61	9.3	0.44	ns	<0.001
Carcass	21.9	21.6	22.6	1.38	Ns	16.9^a^	14.6^b^	14.4^b^	0.54	<0.01	<0.001
Protein content, g/100 g tissue											
Proximal jejunum mucosa	11.3	10.2	10.2	0.44	Ns	11.2	12.0	12.7	0.36	ns	<0.05
Distal ileum mucosa	9.0	8.9	8.7	0.49	Ns	8.8	9.4	8.9	0.40	ns	ns
Liver	12.1	11.7	11.9	0.62	Ns	11.9^a^	14.2^b^	13.7^b^	0.42	<0.05	<0.01
Semitendinosus muscle	7.7	7.9	7.1	0.53	Ns	8.1	9.7	8.5	1.15	ns	ns
Carcass	8.3	8.5	8.7	0.62	Ns	11.4	12.7	11.6	0.59	ns	<0.001

Values are least square means and pooled SEM calculated in pigs slaughtered at d7 and d28, *n* = 6/group. *P* (effect of formula) and age effects are presented. Differences were considered significant when *P* < 0.05 but trends are presented when 0.1 < *P* < 0.05. Means with a different superscript letter differ at *P* < 0.05.

**Table 6:** Proteasome, calpain, and cathepsin L activities (RFU/min/mg protein) measured in various tissues of piglets fed NP, HP, and HPA diets at d7 and d28.

			d7					d28			Age effect
	NP	HP	HPA	SEM	*P*	NP	HP	HPA	SEM	*P*	
Proteasome activity, RFU/min/mg protein											
Proximal jejunum mucosa	25908	25569	26586	718	ns	31343	25827	26137	2445	ns	ns
Distal ileum mucosa	21828	23123	21803	1592	ns	26196	23846	22263	3266	ns	ns
Liver	12499	12848	12767	662	ns	13352^a^	11808^b^	11117^b^	647	0.07	ns
Semitendinosus muscle	634	783	691	56	ns	497	376	578	67	ns	<0.001
Calpain activity, RFU/min/mg protein											
Proximal jejunum mucosa	6469	6842	6794	647	ns	9307	8640	9460	925	ns	<0.001
Distal ileum mucosa	8944	9962	7819	1588	ns	12427	14015	14552	2792	ns	0.02
Liver	11505	11649	12054	914	ns	11045^a^	7451^b^	6884^b^	948	0.01	<0.001
Semitendinosus muscle	529	505	561	92	ns	382	308	524	69	ns	0.06
Cathepsin L activity, RFU/min/mg protein											
Proximal jejunum mucosa	1286	989	1136	393	ns	2511	2943	4125	978	ns	<0.01
Distal ileum mucosa	3041	2971	5642	1091	ns	2965	3019	6085	1127	ns	ns
Liver	2333	3358	2415	609	ns	3889	2301	2530	868	ns	ns
Semitendinosus muscle	965	2237	1068	113	ns	732	572	632	109	ns	<0.001

Values are least square means and pooled SEM calculated in pigs slaughtered at d7 and d28, *n* = 6/group. *P* (effect of formula) and age effects are presented. Differences were considered significant when *P* < 0.05 but trends are presented when 0.1 < *P* < 0.05. Means with a different superscript letter differ at *P* < 0.05.

**Table 7:** Plasma amino acid and urea concentrations measured in piglets fed NP, HP, and HPA diets at d7 and d28.

			d7					d28			Age effect
	NP	HP	HPA	*P*	SEM	NP	HP	HPA	*P*	SEM	
Amino acids, *μ*mol/L											
Lysine	106.4^a^	200.3^b^	188.3^b^	<0.05	26.23	198.2^a^	336.9^b^	306.9^b^	<0.01	28.15	<0.001
Threonine	382.7^a^	749.8^b^	746.8^b^	<0.1	132.26	470.9	583.5	578.6	ns	61.73	ns
Methionine	34.2^a^	66.0^b^	83.7^b^	<0.05	11.31	91.1	78.2	72.7	ns	12.69	<0.05
Tryptophane	40.3^a^	59.6^ab^	71.6^b^	<0.1	8.63	85.9	88.6	98.2	ns	8.98	<0.001
Leucine	205.1^a^	299.1^b^	310.1^b^	<0.1	33.95	274.5	336.8	329.3	ns	23.64	<0.1
Valine	408.1^a^	519.6^b^	498.9^ab^	<0.1	34.81	497.5	492.2	491.6	ns	25.91	ns
Isoleucine	153.3^a^	226.1^b^	225.9^b^	<0.05	21.45	195.3	237.6	234.8	ns	18.04	ns
Phenylalanine	91.6	131.3	158.6	ns	22.15	97.9	118.2	119.6	ns	14.79	ns
Histidine	93.2	108.6	115.2	ns	18.46	143.2^a^	104.7^b^	128.2^ab^	<0.1	11.55	ns
Arginine	118.4^a^	172.6^b^	227.2^b^	<0.05	28.3	187.1	152.6	155.6	ns	30.50	ns
Alanine	649.7	695.5	660.2	ns	59.08	548.9	505.7	448.2	ns	44.11	<0.001
Asparagine	44.6	83.1	78.5	ns	16.80	77.6	72.9	72.7	ns	9.48	ns
Aspartic acid	42.1	71.7	69.2	ns	12.00	42.7	57.8	63.5	ns	9.03	ns
Citrulline	137.9	144.6	151.1	ns	14.78	214.4	142.5	167.7	ns	26.56	<0.1
Cystine	37.6	47.4	56.1	ns	8.63	34.3	21.3	20.9	ns	4.81	<0.001
Glutamine	424.7	531.5	559.8	ns	75.32	516.0	431.8	423.1	ns	37.67	ns
Glutamic acid	247.6	297.2	266.1	ns	36.29	332.5^a^	249.1^b^	228.1^b^	<0.05	24.54	ns
Glycine	1250.7	1370.6	1444.1	ns	116.60	2182.7^a^	1315.2^b^	1291.9^b^	<0.01	168.3	<0.05
Serine	245.2^a^	311.4^b^	336.1^b^	<0.1	28.57	322.6^a^	230.4^b^	227.0^b^	<0.05	25.49	<0.1
Ornithine	81.8	91.4	84.3	ns	6.12	166.7	152.9	173.5	ns	18.29	<0.001
Proline	712.3	860.7	816.2	ns	58.02	786.4	625.7	638.7	ns	91.43	<0.1
Taurine	206.7	269.1	341.6	ns	48.88	110.4^a^	234.6^b^	242.7^b^	<0.01	24.89	<0.05
Tyrosine	125.5	220.4	240.6	ns	39.06	151.1	153.4	173.1	ns	19.55	ns
NH_3_, *μ*mol/L	368.0	340.0	337.2	ns	38.66	379.9^a^	486.8^b^	476.9^b^	<0.05	28.62	<0.001

Values are least square means and pooled SEM calculated in pigs slaughtered at d7 and d28, *n* = 6/group. *P* (effect of formula) and age effect are presented. Differences were considered significant when *P* < 0.05 but trends are presented when 0.1 < *P* < 0.05. Means with a different superscript letter differ at *P* < 0.05.

## References

[1] Thureen P, Heird WC (2005). Protein and energy requirements of the preterm/low birthweight (LBW) infant. *Pediatric Research*.

[2] Frank JW, Escobar J, Suryawan A (2005). Protein synthesis and translation initiation factor activation in neonatal pigs fed increasing levels of dietary protein. *Journal of Nutrition*.

[3] Gras-Le Guen C, Boscher C, Godon N (2007). Therapeutic amoxicillin levels achieved with oral administration in term neonates. *European Journal of Clinical Pharmacology*.

[4] Chatelais L, Jamin A, Guen CG-L, Lallès J-P, le Huërou-Luron I, Boudry G (2011). The level of protein in milk formula modifies ileal sensitivity to LPS later in life in a piglet model. *PLoS One*.

[5] Bauer R, Walter B, Hoppe A (1998). Body weight distribution and organ size in newborn swine (*sus scrofa domestica*)—a study describing an animal model for asymmetrical intrauterine growth retardation. *Experimental and Toxicologic Pathology*.

[6] Burke C, Sinclair K, Cowin G (2006). Intrauterine growth restriction due to uteroplacental vascular insufficiency leads to increased hypoxia-induced cerebral apoptosis in newborn piglets. *Brain Research*.

[7] Bauer R, Walter B, Brust P, Füchtner F, Zwiener U (2003). Impact of asymmetric intrauterine growth restriction on organ function in newborn piglets. *European Journal of Obstetrics Gynecology & Reproductive Biology*.

[8] Rehfeldt C, Kuhn G (2006). Consequences of birth weight for postnatal growth performance and carcass quality in pigs as related to myogenesis. *Journal of animal science*.

[9] Wu G, Ott TL, Knabe DA, Bazer FW (1999). Amino acid composition of the fetal pig. *Journal of Nutrition*.

[10] Quesnel H, Brossard L, Valancogne A, Quiniou N (2008). Influence of some sow characteristics on within-litter variation of piglet birth weight. *Animal*.

[11] Dourmad JY, Noblet J, Etienne M (1998). Effect of protein and lysine supply on performance, nitrogen balance, and body composition changes of sows during lactation. *Journal of Animal Science*.

[12] Klobasa F, Werhahn E, Butler JE (1987). Composition of sow milk during lactation. *Journal of Animal Science*.

[13] Jamin A, D’Inca R, Le Floc’H N (2010). Fatal effects of a neonatal high-protein diet in low-birth-weight piglets used as a model of intrauterine growth restriction. *Neonatology*.

[14] Sève B, Ballèvre O, Ganier P, Noblet J, Prugnaud J, Obled C (1993). Recombinant porcine somatotropin and dietary protein enhance protein synthesis in growing pigs. *Journal of Nutrition*.

[15] Hamard A, Seve B, Le Floc’h N (2009). A moderate threonine deficiency differently affects protein metabolism in tissues of early-weaned piglets. *Comparative Biochemistry and Physiology, Part A*.

[16] Lowry OH, Rosebrough NJ, Farr AL, Randall RJ (1951). Protein measurement with the Folin phenol reagent. *The Journal of Biological Chemistry*.

[17] Leblond J, Hubert-Buron A, Bole-Feysot C, Ducrotte P, Dechelotte P, Coeffier M (2006). Regulation of proteolysis by cytokines in the human intestinal epithelial cell line HCT-8: role of IFNgamma. *Biochimie*.

[18] Bradford MM (1976). A rapid and sensitive method for the quantitation of microgram quantities of protein utilizing the principle of protein dye binding. *Analytical Biochemistry*.

[19] Suryawan A, O’Connor PMJ, Bush JA, Nguyen HV, Davis TA (2009). Differential regulation of protein synthesis by amino acids and insulin in peripheral and visceral tissues of neonatal pigs. *Amino Acids*.

[20] Davis TA, Fiorotto ML, Burrin DG (2002). Stimulation of protein synthesis by both insulin and amino acids is unique to skeletal muscle in neonatal pigs. *American Journal of Physiology*.

[21] Adegoke OA, McBurney MI, Samuels SE, Baracos VE (2003). Modulation of intestinal protein synthesis and protease mRNA by luminal and systemic nutrients. *The American Journal of Physiology*.

[22] Hamard A, Mazurais D, Boudry G, Le Huërou-Luron I, Sève B, Le Floc’h N (2010). A moderate threonine deficiency affects gene expression profile, paracellular permeability and glucose absorption capacity in the ileum of piglets. *Journal of Nutritional Biochemistry*.

[23] Li Z, Cao B, Zhao B, Yang X, Fan MZ, Yang J (2009). Decreased expression of calpain and calpastatin mRNA during development is highly correlated with muscle protein accumulation in neonatal pigs. *Comparative Biochemistry and Physiology, Part A*.

[24] Wang J, Chen L, Li D (2008). Intrauterine growth restriction affects the proteomes of the small intestine, liver, and skeletal muscle in newborn pigs. *Journal of Nutrition*.

[25] Bechet D, Tassa A, Taillandier D, Combaret L, Attaix D (2005). Lysosomal proteolysis in skeletal muscle. *International Journal of Biochemistry and Cell Biology*.

[26] Boukhettala N, Leblond J, Claeyssens S (2009). Methotrexate induces intestinal mucositis and alters gut protein metabolism independently of reduced food intake. *The American Journal of Physiology*.

[27] Potter DA, Srirangam A, Fiacco KA (2003). Calpain regulates enterocyte brush border actin assembly and pathogenic Escherichia coli-mediated effacement. *Journal of Biological Chemistry*.

[28] Ekstrom GM, Westrom BR (1991). Cathepsin B and D activities in intestinal mucosa during postnatal development in pigs. Relation to intestinal uptake and transmission of macromolecules. *Biology of the Neonate*.

[29] Kadowaki M, Kanazawa T (2003). Amino acids as regulators of proteolysis. *Journal of Nutrition*.

[30] Raiha NC, Heinonen K, Rassin DK, Gaull GE (1976). Milk protein quantity and quality in low birthweight infants: I. Metabolic responses and effects on growth. *Pediatrics*.

[31] Kalhan SC, Bier DM (2008). Protein and amino acid metabolism in the human newborn. *Annual Review of Nutrition*.

[32] Sarr O, Gondret F, Jamin A, Le Huërou-Luron I, Louveau I (2011). A high-protein neonatal formula induces a temporary reduction of adiposity and changes later adipocyte physiology. *The American Journal of Physiology*.

[33] Shen Q, Xu H, Wei LM, Chen J, Liu HM (2010). Intrauterine growth restriction and postnatal high-protein diet affect the kidneys in adult rats. *Nutrition*.

[34] Poore KR, Fowden AL (2004). The effects of birth weight and postnatal growth patterns on fat depth and plasma leptin concentrations in juvenile and adult pigs. *Journal of Physiology*.

[35] Patel MS, Srinivasan M (2002). Metabolic programming: causes and consequences. *Journal of Biological Chemistry*.

